# A pulmonary ligament approach for portal robotic segmentectomy of the lateral and posterior basal segments: a case report

**DOI:** 10.1186/s13256-021-02789-3

**Published:** 2021-04-25

**Authors:** Shota Mitsuboshi, Hiroaki Shidei, Akihiro Koen, Hideyuki Maeda, Hiroe Aoshima, Tamami Isaka, Masato Kanzaki

**Affiliations:** Department of Thoracic Surgery, Tokyo Women’s Medical University, 8-1 Kawada-cho, Shinjuku-ku, Tokyo, 162-8666 Japan

**Keywords:** Robot-assisted thoracoscopic surgery, Segmentectomy, Pulmonary ligament, Thoracoscopy, Intersegmental septum

## Abstract

**Background:**

Thoracoscopic segmentectomy of the lateral and posterior basal segments is extremely technically challenging. Appropriate segmentectomy requires exposure and recognition of the branches of the bronchi and pulmonary vessels deep in the lung parenchyma. Although various approaches for these segmentectomies have been reported, the use of a pulmonary ligament approach is rational because it does not require any interlobar separation. Here, we report a successful case of portal robotic segmentectomy of the lateral and posterior basal segments through the pulmonary ligament approach.

**Case presentation:**

A 60-year-old Japanese man with a history of low anterior resection for rectal cancer was referred to our department because of a lung nodule. His chest computed tomography revealed a 15-mm tumor in the left posterior basal bronchus. Robotic left S9–10 segmentectomy through the pulmonary ligament was performed with five-port incisions.

**Conclusions:**

An extremely technically challenging thoracoscopic segmentectomy of the lateral and posterior basal segments was performed through the pulmonary ligament using a robotic surgical system.

**Supplementary Information:**

The online version contains supplementary material available at https://doi.org/10.1186/s13256-021-02789-3.

## Background

In Japan, the national health insurance began covering robotic pulmonary segmentectomy for malignant lung tumors in 2020, in consideration of the increasing number of domestic robot-assisted thoracoscopic surgical (RATS) procedures being performed. Recently, robotic segmentectomy has been reported to have been performed safely and have excellent perioperative outcome and safety [1–3].

However, thoracoscopic segmentectomy of the lateral (S9) and posterior basal (S10) segments is extremely technically challenging. Appropriate segmentectomy requires exposure and recognition of the branches of the bronchi and pulmonary vessels deep in the lung parenchyma [1–3]. The branches of the pulmonary vein are especially far from the interlobar fissure. Although various approaches for these segmentectomies have been reported, the use of a pulmonary ligament (PL) approach is rational because it does not require any interlobar separation.

Here, we report successful case of portal robotic S9 and S10 (S9–10) segmentectomy through the PL approach.

## Case presentation

**Fig. 1:**
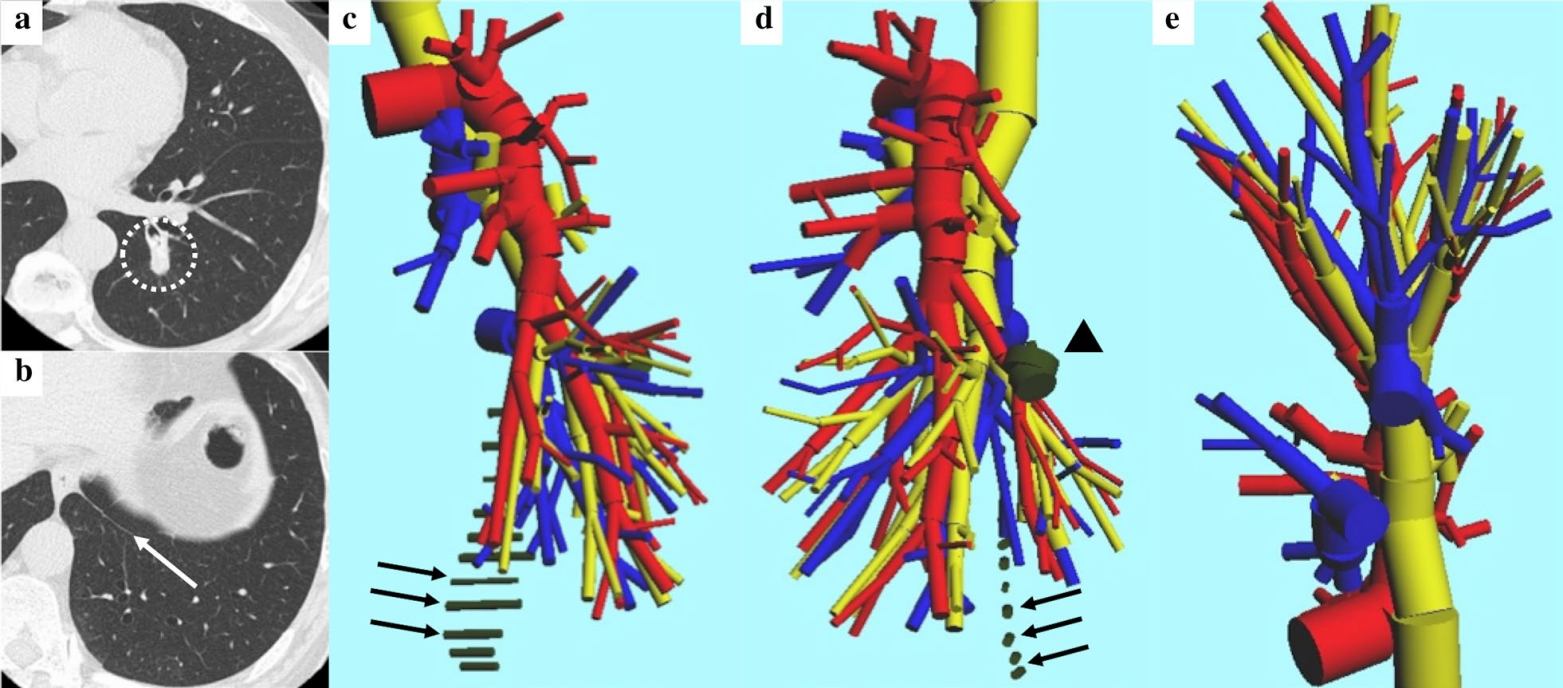
**a**, **b** Chest computed tomography revealing a 15-mm tumor in the left posterior basal bronchus (dotted circle) and the pulmonary ligament (white arrow). **c**–**e** Preparation of a reconstructed 3-D pulmonary model of patients with metastatic lung tumor. The red color indicates the pulmonary arteries, blue color indicates the pulmonary veins, yellow color indicates the trachea and bronchi, and black color indicates the tumor (arrow head) and the pulmonary ligament (arrows).

A 60-year-old Japanese man with a history of low anterior resection for rectal cancer 4 years ago was referred to our department because of a lung nodule detected on chest radiography during a routine medical checkup. He had no symptoms and presented with a body temperature of 35.9°C, blood pressure of 106/65 mmHg, heart rate of 59 beats per minute, and oxygen saturation of 99% on room air. His laboratory examination results were within normal limits. As his chest computed tomography (CT) revealed a 12-mm tumor in the left posterior basal bronchus during postoperative follow-up, he underwent six cycles of chemotherapy with mFOLFOX6 and bevacizumab at standard doses. After chemotherapy, he underwent chest and abdomen CT scans, which confirmed that the tumor deeply seated in the segment had been growing continuously up to 15 mm (Fig. 1a, b). Fluorodeoxyglucose positron emission tomography (FDG-PET) revealed an abnormal uptake of FDG, with a maximum standardized uptake value of 2.28 in the tumor. Hence, the patient was treated with a portal robotic S9–10 segmentectomy through a PL approach.

On the basis of the patient’s actual three-dimensional (3-D) pulmonary model, created using an in-house software as presented in the preoperative CT images, the involvement of the pulmonary vessels and bronchi were identified and the location and extent of tumor invasion were assessed to determine the surgical procedure (Fig. 1c–e) [4–6].

**Fig. 2:**
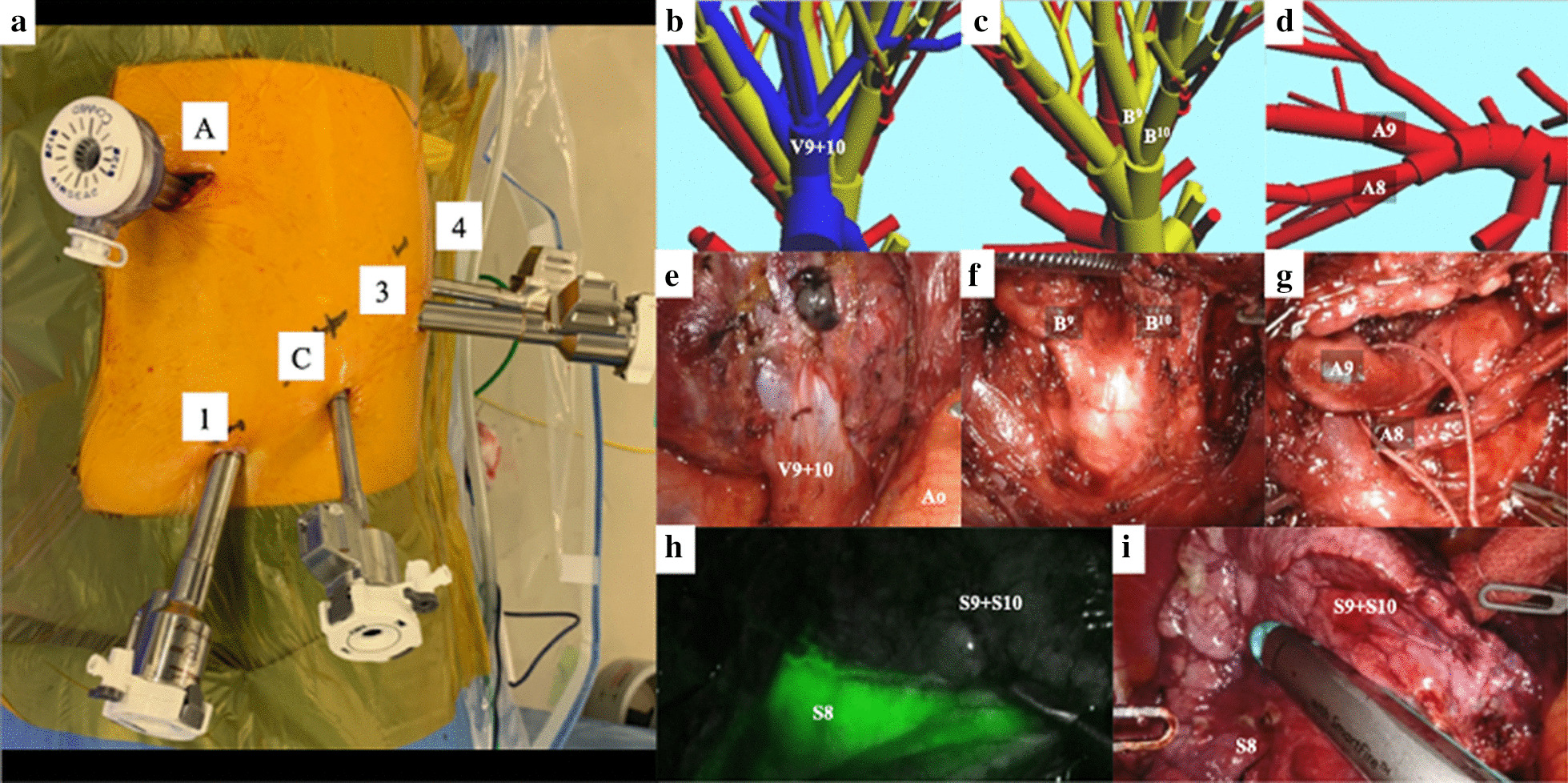
**a** Port placement for robotic left S9–10 segmentectomy through a pulmonary ligament approach. *A* assistant port, *1* port 1, *C* camera port, *3* port 3, *4* port 4. **b**–**g** Comparison of the actual surgical field of vision and the three-dimensional model for a patient. **b**, **e** The lateral basal vein (V9) and posterior basal vein (V10) are exposed. **c**, **f** After the V9 and V10 were transected and the dissection was performed along the intersegmental septum, the lateral basal bronchus (B^9^) and posterior basal bronchus (B^10^) were exposed. **d**, **g** After the B^9^ and B^10^ were transected, the lateral basal artery (A9) and posterior basal artery (A10) were exposed and transected. **h** Intersegmental planes were identified with an intravenous injection of indocyanine green dye. **i** The identified intersegmental planes were transected by robot staplers. *A8* anterior basal artery, *Ao* aorta, *S8* anterior basal segment.

Under general anesthesia with single-lung ventilation and lateral decubitus positioning, RATS S9–10 segmentectomy was performed with five-port incisions, including an assistant port as a carbon dioxide (CO_2_) insufflation port. With the pleural space as the entry point, a 12-mm trocar (AirSeal access ports, ConMed, Utica, NY, USA) was inserted as an assistant port in the fifth intercostal space (ICS) anteriorly in the anterior axillary line. Moreover, two 8-mm robotic trocars were inserted, one as a port for the robotic camera in the ninth ICS at the middle axillary line and the other as port 4 on the posterior side of the tip of the scapula. Two 12-mm robotic trocars were inserted in port 1 and 3 in the eighth ICS anteriorly along the anterior axillary line and in the ninth ICS along the posterior axillary line, respectively, after which the da Vinci Xi surgical system (Intuitive Surgery, Sunnyvale, CA, USA) was docked (Fig. 2a). All four robotic arms were used. A CO_2_ insufflation system (AirSeal system, ConMed) was used at a set pressure of 5 mmHg. The robotic instruments were manipulated through a 12-mm port mounting a 12–8-mm reducer. Fenestrated bipolar forceps, a permanent cautery spatula, and Cadiere forceps were inserted through ports 1, 3, and 4 (Intuitive Surgical), respectively. After lifting the left lower lobe using the Cadiere forceps, the PL was incised up to the inferior pulmonary vein. The basal pulmonary vein was exposed, and both the lateral (V9) and posterior basal veins (V10) were transected using robot staplers (Fig. 2b, e). Next, the intersegmental septum was dissected to expose the bronchi and pulmonary arteries. First, the targeted bronchi were exposed and transected, followed by the targeted pulmonary arteries (Fig. 2c, d, f, g). An intravenous injection of indocyanine green was administered, and observation under fluorescence navigation revealed intersegmental planes, which were marked using the fenestrated bipolar forceps and permanent cautery spatula, after which the target S9–10 segments were resected using the robot staplers (Fig. 2h, i). An additional movie file shows this procedure in detail (Additional file 1).

The final histopathological investigations confirmed the resected tumor to be a lung metastasis of rectal cancer. The postoperative course was uneventful. Postoperatively, he received no adjuvant chemotherapy. No recurrence was observed for 6 months after the operation.

## Discussion and conclusions

Since March 2009, we have performed a complete thoracoscopic segmentectomy of S9–10 through a PL approach while viewing two-dimensional (2-D) images on a video monitor in 37 patients with primary lung cancer and metastatic lung tumors [4]. The PL is contiguous with the intersegmental septum and can be easily surgically separated from the lung parenchyma, such as the medial basal segment (S7) and S10 of the right lung and the anterior basal segment (S8) and S10 of the left lung. Thus, interlobar separation of the lungs is not mandatory to expose the inferior pulmonary vein. By allowing accurate thoracoscopic treatment of the peripheral vessels and the segmental bronchi, 3-D images can be used to visualize the positional relationships among the target pulmonary vessels and bronchi [7, 8]. In comparison with the conventional 2-D thoracoscopic S9 or S10 segmentectomy through a PL approach, portal robotic S9–10 segmentectomy is feasible. The use of robotic surgical systems yields accurate 3-D high-definition images with improved ergonomics that allows surgeons to conduct accurate and improved intuitive maneuvers, which reduces the burden on the assistant surgeon. Compared with the conventional 2-D thoracoscopic surgery through a PL approach, multijointed robotic forceps allow easy mobilization on the diaphragmatic surface of the inferior lobe to the cranial side and easy dissection of the intersegmental septum to expose the pulmonary vessels and bronchi in the RATS procedure. While our previously reported video-assisted thoracoscopic surgical (VATS) technology may confer a higher risk of incomplete lymph node dissection than the traditional procedures would, the RATS technique articulates lymphadenectomy, which is difficult to execute with thoracoscopy and can be easily performed with robotic instruments [9]. RATS removes the physiological tremor known in VATS. This results in an excellent level of precision necessary for such a challenging case [10]. Early-stage lung cancer, metastatic lung tumors, and benign tumors arising in the lung center are considered good indications for the RATS technique. Furthermore, the learning curve for RATS is reported to be shorter than that for thoracoscopic surgery [9]. Although S9–10 segmentectomy thorough the PL approach is extremely technically challenging, the RATS technique is considered faster to master.

## Supplementary Information

**Additional file 1.** After the left lower lobe was lifted up using Cadiere forceps, the pulmonary ligament was incised up to the inferior pulmonary vein. The basal pulmonary vein was exposed, and both the lateral basal vein and posterior basal vein were transected using robot staplers. Next, the intersegmental septum was dissected to expose the bronchi and pulmonary arteries. The targeted bronchi, such as the lateral basal bronchus and posterior basal bronchus, were first exposed and transected, followed by the targeted pulmonary arteries as the lateral basal artery and posterior basal artery. An intravenous injection of indocyanine green was administered, and observation under fluorescence navigation revealed intersegmental planes, which were marked using fenestrated bipolar forceps and permanent cautery spatula, after which the target S9–10 segments were resected using robot staplers.

## Data Availability

All the data and materials supporting our findings are included within the article.

## Abbreviations

RATS: Robot-assisted thoracoscopic surgical
S9: Lateral segment
S10: Posterior basal segment
PL: Pulmonary ligament
S9–10: Lateral and posterior basal segments
3-D: Three-dimensional
CT: Computed tomography
FDG-PET: Fluorodeoxyglucose positron emission tomography
CO_2_: Carbon dioxide
ICS: Intercostal space
V9: Lateral veins
V10: Posterior basal veins
2-D: Two-dimensional
S7: Medial basal segment
S8: Anterior basal segment
VATS: Video-assisted thoracoscopic surgical
